# A Review of Intentional and Cognitive Control in Autism

**DOI:** 10.3389/fpsyg.2012.00436

**Published:** 2012-10-25

**Authors:** Edita Poljac, Harold Bekkering

**Affiliations:** ^1^Department of Experimental Psychology, University of OxfordOxford, UK; ^2^Donders Institute for Brain, Cognition and Behaviour, Radboud University NijmegenNijmegen, Netherlands

**Keywords:** autism, cognitive control, intentions, actions, behavioral rigidity, open and closed systems

## Abstract

Different clinical studies have provided empirical evidence for impairments in cognitive control in individuals with autism spectrum disorders (ASD). The challenge arises, however, when trying to specify the neurocognitive mechanisms behind the reported observations of deviant patterns of goal-directed behavior in ASD. Studies trying to test specific assumptions by applying designs that are based on a more controlled experimental conditions often fail in providing strong evidence for an impairment in specific cognitive functions. In this review, we summarize and critically reflect on behavioral findings and their theoretical explanations regarding cognitive control processing in autism, also from a developmental perspective. The specific focus of this review is the recent evidence of deficits in intentional control – a specific subset of cognitive control processes that biases the choice of our behavioral goals – coming from different research fields. We relate this evidence to the cognitive rigidity observed in ASD and argue that individuals with ASD experience problems at the intentional level rather than at the level of implementation of intentions. Both these processes are related to cognitive control mechanisms but in different ways. Finally, we discuss new directions in studying cognitive control in ASD and how these relate to adaptive cognition.

## Introduction to Autism

Autism is a neurodevelopmental disorder characterized by severe disturbances in reciprocal social relations and varying degrees of language and communication difficulties (American Psychiatric Association, 1994). In addition to these social and communication problems, individuals with autism tend to exhibit a preference for sameness, often reflected in behavioral patterns and interests that are restricted and repetitive. In this paper we will focus on possible deficits in intentional control and relate these to the rigidity observed in autism.

This pervasive developmental disorder (PDD) manifests at early age, with a wide range in symptoms varying greatly across age and abilities (McBride et al., 1996). To recognize this diversity, the notion of autism spectrum has been proposed (Folstein and Rosen-Scheidley, 2001), referring to both the extensive variety in functional abilities across the affected individuals and to the set of relatively heterogeneous disorders that make up the autism category. Autism spectrum disorders (ASD) include Autistic Disorder, Asperger’s Disorder, and PDD-Not Otherwise Specified (PDD-NOS). In fact, the autism spectrum has been expanded even further by different clinical and experimental studies reporting sub-clinical autistic symptomatology in family members of individuals with ASD (Eisenberg, 1957; Fombonne et al., 1997; Pickles et al., 2000). The realization of the broader autism phenotype (BAP) has motivated further experimental studies on parents and siblings of children with ASD (Gerdts and Bernier, 2011) and a large amount of studies with a genetic approach (for a recent review on BAP, see Sucksmith et al., 2011).

It is not surprising then that grasping such a complex disorder as ASD through a general framework that describes the neurocognitive impairments underlying ASD is an enormous challenge to the scientific community. Great efforts have been made so far to understand ASD in terms of its expression in overt behavior and brain structure and activity (see Belger et al., 2011; Stigler et al., 2011; Just et al., 2012; Philip et al., 2012;, for recent reviews on neuroimaging in autism), its biological origin, and the specific genetic and environmental factors that might be involved (Eigsti and Shapiro, 2003; Geschwind, 2011). Different cognitive theories have been proposed to account for certain aspects of the observed behavioral symptoms. According to an increasingly influential framework, some of the observed behavioral symptoms in ASD – like for instance the often reported rigidity in behavior – are generated by deficits in cognitive control (Russell, 1997; Hill, 2004a,b). The specific contribution of the current review is to summarize behavioral findings and their theoretical explanations regarding cognitive control processing in ASD and to critically reflect on both. Although neuroimaging data are beyond the scope of this review, we will refer to the most relevant (review) studies from neuroscience throughout the text. After a brief introduction on cognitive control mechanisms, we will review the relevant clinical and experimental literature from the research field on task switching, and on intentions and actions in ASD. We will in particular examine the recent evidence of deficits in intentional control coming from different research fields and relate this evidence to the rigidity observed in ASD. We refer to intentional control as a specific subset of cognitive control processes that biases the choice of our behavioral goals and hence further facilitates selection and monitoring of goal-directed actions (cf. Mayr and Bell, 2006; Butler et al., 2011). We will also reflect on findings about cognitive control mechanisms across different age groups. Finally, we will discuss new directions in studying cognitive control in ASD and their relation to adaptive cognition.

## Cognitive Control in ASD

### What is cognitive control?

Understanding how people guide their thoughts and actions is a long-standing challenge in psychology and neuroscience. Being able to behave in accordance with current intentions is suggested to depend on dedicated neurocognitive control mechanisms (Norman and Shallice, 1986; Miller and Cohen, 2001). These control mechanisms allow us to sustain focus on the information relevant to the behavioral goal we wish to achieve while competing with possible distractions, and to change focus when required. In other words, cognitive control allows for goal-directed and flexible behavior in a dynamically changing environment. When this top-down control fails – due to some temporary distraction or a permanent deficit – behavior is expected to be governed by habitual or recently activated pathways. Even with top-down input, performance is expected to be less efficient if our current behavioral goals conflict with habitual patterns of behavior.

Processes involved in cognitive control have often been investigated experimentally by asking people to switch between different cognitive tasks (Sakai, 2008; Kiesel et al., 2010; Vandierendonck et al., 2010). Participants would for instance be required to switch between responding to the color and responding to the shape of geometric figures. In these well-established task switching paradigms, tasks are mostly specified by a task cue provided on each experimental trial or by predefined task orders. The large amount of studies conducted within this research field has provided empirical evidence that people are indeed able to switch quickly and flexibly from one task to another. People, however, experience cognitive limitations while doing so: they slow down and make more errors when switching (Allport et al., 1994; Rogers and Monsell, 1995; Meiran, 1996; Rubinstein et al., 2001). Surprisingly, this switch cost is reduced but not abolished when providing ample time to prepare for the required switch (Rogers and Monsell, 1995; Meiran, 2000; Mayr and Kliegl, 2003; Poljac et al., 2006). The ability to switch tasks as well as the reduction of switch costs with ample preparation time are typically taken as a clear expression of top-down intentional control, whereas the residual cost is attributed to different bottom-up effects disrupting goal-directed behavior. So far, a broad consensus has been reached that performance in instructed task switching experiments reflects a complex interaction between top-down control and bottom-up interference (Kiesel et al., 2010; Vandierendonck et al., 2010).

Besides task switching, updating of working memory contents and inhibition of interfering information have also usually been identified as important contributors to the control of thought and action (Miyake et al., 2000; Miyake and Friedman, 2012; for neuroimaging findings, see Berkman et al., 2012). Updating refers to the ability to monitor incoming information and to adjust the content of working memory according to the current behavioral goal. Inhibition, on the other hand, is often proposed as a process that resolves interference from competing thoughts and actions (Friedman and Miyake, 2004), and as such allows for appropriate decision making (for a different view on action control, see e.g., Allport, 1987). Inhibitory control is a strongly debated topic in the literature. The debate goes, for instance, from questions about the meaning of the concept in the study of cognition and neuroscience (MacLeod, 2007) to how unitary the concept is (Friedman and Miyake, 2004; Munakata et al., 2011) and what its functional role might be in serial-task control (Mayr, 2009; Koch et al., 2010) or memory (Levy and Anderson, 2002). Inhibition as an active and quick cognitive process is suggested to be closely related to interference (Friedman and Miyake, 2004), but so is passive and more persistent decay of activation (Altmann and Schunn, 2012).

Although the exact functional role of inhibition and its relation to interference, decay, and cognitive control needs to be more clearly specified, the existence of inhibition and its modulation of behavior has been shown empirically by various studies. These studies usually apply fairly simple cognitive tasks – such as Go/No Go and stop-signal tasks – developed to assess inhibitory processes, typically focusing on response inhibition (Logan, 1994). The participant is for instance required to quickly respond to some stimulus material (e.g., pressing a button when seeing the letters Q, P, T) but to inhibit responding either to specific stimulus (e.g., to the letter X) or when hearing auditory input (e.g., when a beep is sounded). Behavioral index of inhibitory control is the number of errors made or the time it takes to stop responding (see Aron, 2007 for a review on neuroscience findings). Similar to tasks investing inhibitory control, different experimental tasks are developed for investigating the updating ability, such as for instance, the letter memory and the n-back tasks. In the letter memory task, participants are required to remember the last four letters of a series of sequentially presented letters and to rehears them out loud throughout the task. In the n-back task, participants would typically be asked to indicate whether each stimulus in a sequence matches the one that appeared on n trials before. Behavioral index of updating in these tasks is the number of letters recalled incorrectly or trials with incorrectly indicated stimulus dimension match, and the speed with which the latter is done (for a recent study on neural correlates of updating, see Nee and Brown, 2012).

### Executive dysfunction approach in ASD

Investigating cognitive processes that allow for goal-directed behavior in clinical populations and hence individuals with ASD is often done by focusing on executive functions. These cognitive functions relate to the higher-order neurocognitive control processes and encompass for instance initiation and monitoring of actions, mental flexibility, planning, working memory, impulse control, and inhibition (Rabbitt, 1997a; Roberts et al., 1998; Miyake et al., 2000; Stuss and Knight, 2002). Executive functions have traditionally been linked to frontal lobes (Miller and D’Esposito, 2005; Seniów, 2012), although this relation is not necessarily a direct one (Alvarez and Emory, 2006). Moreover, involvement of the parietal cortex (Stoet and Snyder, 2009; De Baene et al., 2012) and other brain areas has recently become evident (see Funahashi, 2001; Jurado and Rosselli, 2007 for a review), suggesting a more distributed nature of the neural correlates of executive functions.

Deficits in executive functions have been associated with some of the everyday social behaviors seen in individuals with ASD (Ozonoff et al., 1991; Happé et al., 2006). Interestingly, White (2012) has recently proposed that poor performance measured in individuals with ASD on tasks developed to assess executive functions originates from difficulties they experience with understanding the experimenter’s expectations for these often ambiguously defined tasks rather than from their executive dysfunction. According to White, deviant scores on these tasks are best understood as a reflection of difficulties in forming an implicit understanding of expectations of others and problems with taking another’s perspective, assumed to be the case in ASD (see Boucher, 2012 for a recent critical review). Executive dysfunction is, however, mostly related to non-social behaviors in ASD, such as a need for sameness, a strong tendency for repetitive behaviors, lack of impulse control, and difficulties in switching between tasks (Hill, 2004a,b; Rajendran and Mitchell, 2007; for frontal lobes abnormalities in ASD, see Schmitz et al., 2007).

Usually deficits in executive functions are detected by means of different neuropsychological tests. Various studies using these tests have reported different executive impairments in ASD, such as deficits in planning (Hughes et al., 1994; Ozonoff and McEvoy, 1994; Hughes, 1996; Ozonoff and Jensen, 1999; Geurts et al., 2004; Ozonoff et al., 2004; Hill and Bird, 2006), the inability to generate novel ideas and behaviors spontaneously (Boucher, 1988; Craig and Baron-Cohen, 1990; Turner, 1999; Wong et al., 2003), selective inhibitory impairments (Christ et al., 2007, 2011; Adams and Jarrold, 2012), and problems with cognitive flexibility (Ozonoff, 1997; Shu et al., 2001; Ozonoff et al., 2004; Zelazo, 2006). For instance, difficulties with planning in individuals with ASD become evident when being tested on a task commonly used to assess planning and problem solving skills in patients – the Tower of London (ToL) task (Ozonoff and McEvoy, 1994; Robinson et al., 2009). In this task, participants are presented with a prearranged sequence of different colored beads on different sized pegs. They are required to move the beads such to match a predetermined goal state shown on a parallel board of pegs determined by the examiner. The instruction given is to do so in as few moves as possible, while obeying some prespecified rules. Robinson and colleagues demonstrated that children with ASD needed more moves to solve these problems and that they violated the rules of the task more often than typically developing children (see Just et al., 2007 for the neural bases of the deviant performance on ToL in ASD). In the same study, the authors observed furthermore difficulties with inhibition of proponent responses (for neuroimaging data, see Kana et al., 2007). The task used was a computerized version of the Stroop task, with color words printed in different color ink. Children were required to indicate the color of the ink that the word was written in. The findings demonstrated that children with ASD were poorer in responding to items in which the color indicated by the word did not match the ink in which the word was written (e.g., word “green” printed in blue ink). Altogether, different studies have demonstrated deficits in executive function in individuals with ASD at the behavioral level (for neural correlates, see e.g., Schmitz et al., 2006). Interestingly, empirical evidence has also been provided for executive dysfunction in parents (Hughes et al., 1997) and siblings (Hughes et al., 1999) of children with ASD but not in the BAP (Maes et al., 2012).

### Challenges with the executive dysfunction approach

It is evident that the proposed idea of executive dysfunction in ASD has received empirical support from different studies. The difficulty arises, however, when we try to provide a comprehensible theoretical explanation identifying the neurocognitive mechanisms for the observed empirical findings. First, the idea of executive dysfunction requires further specification in order to be informative and functional for diagnosis, intervention, and theoretical understanding (see Jurado and Rosselli, 2007 for a comprehensive review on executive functions). More precisely, considering the fact that executive functions cover different control mechanisms, a theory in terms of executive dysfunction might apply to almost any neuropsychological disorder without being specifically related to autism. For instance, difficulties in executive functions have also been debated and proposed as a candidate for being a core feature of Gilles de la Tourette Syndrome (GTS, Eddy et al., 2012), attention deficit hyperactivity disorder (ADHD; Willcutt et al., 2005; Brown, 2006; Antshel et al., 2010), schizophrenia (Freedman and Brown, 2011), Parkinson’s disease (PD; Cameron et al., 2012) and other neurodevelopmental disorders. If these various disorders were indeed all based on executive dysfunction, one would expect then that they must share most of their underlying neurocognitive mechanisms. Yet we all agree that these neurodevelopmental disorders differ substantially enough from each other to treat them as being separate disorders. In fact, executive function deficits are not the single necessary and sufficient cause of any of these disorders. So, the danger behind applying such a broad theoretical concept as the core feature of disorders lies in the possibility of false impression that different neuropsychological disorders stem from similar neurocognitive deficits by simply placing these deficits under the same executive functions umbrella.

Second challenge relates more to the measures applied in studies testing executive functions in ASD. Mostly, these are complex neuropsychological tests that put high demands on different cognitive capacities. Clearly, these tests are sensitive enough to detect existing deviations in behavioral patterns in individuals with ASD as compared to typically developing individuals or to some other clinical populations. The complexity of these tests, however, makes it difficult to interpret any observed differences in behavior in a straightforward and intelligible way. The solution, one might think, would be to either break down the commonly used tests to less complex components of executive functions or to isolate the component of interest by making it the only varying factor in the test. The question that arises then is how much of an “executive” is left in such a controlled situation (cf. Burgess, 1997; Rabbitt, 1997b; Brown, 2006). This might perhaps explain why executive abilities in daily life of individuals with ASD as rated by their close relatives and trainers are not inevitably consistent with executive abilities measured by neuropsychological tests (e.g., Chaytor et al., 2006; Teunisse et al., 2012). Furthermore, if the scores on tests developed to assess executive functions reflect the working of the neurocognitive mechanisms responsible for executive abilities, then training people on these tasks should not only improve their performance on the specific task but also on most of other executive tasks, as well as improving how they handle daily activities. This is, however, mostly not the case: trainings seem to produce very specific, short-term effects on the trained task, without transfer of trained skills to the situations outside the training context or long-term retention (Melby-Lervåg and Hulme, 2012).

Recently, Geurts et al. (2009) have addressed a related issue focusing on cognitive flexibility in autism. Cognitive flexibility refers to the ability or a property of the cognitive system to dynamically activate and modify cognitive processes in response to changing task demands and context factors (Deák, 2003; Monsell, 2003; Ionescu, 2012). In their review, Geurts and colleagues give a critical examination of the existing disparity between the strong belief of the clinicians and researchers that cognitive inflexibility (i.e., rigidity) is central to ASD on the one hand, and the lack of consistent unambiguous empirical evidence for this belief on the other. The idea of cognitive inflexibility in ASD has been put forward for at least two different reasons – individuals with ASD exhibit rigid and repetitive behaviors in daily lives, and neuropsychological tests that are developed to scan global individual capacity in mental flexibility have indicated differences in behavioral patterns between typically developing and individuals with ASD. For instance, neuropsychological tests like the Intradimensional/Extradimensional (ID/ED) shift task of the Cambridge Neuropsychological Test Automated Battery (CANTAB; Ozonoff et al., 2004) or the Dimensional Change Card Sort (Zelazo, 2006) have been used to detect differences in cognitive flexibility. The ID/ED task consists of shifts within one stimulus dimension (ID) and between different stimulus dimensions (ED), with the latter considered to reflect cognitive flexibility the most. When assessed in individuals with ASD, the outcomes vary in showing deficits on this task: while some studies have indeed reported impairments in ASD on the ED component (Ozonoff et al., 2004; Yerys et al., 2009; but, see Landa and Goldberg, 2005), the majority of studies find no group differences (Edgin and Pennington, 2005; Goldberg et al., 2005; Happé et al., 2006; Corbett et al., 2009). The test that has most clearly indicated cognitive inflexibility in ASD is the Wisconsin Card Sorting Test (WCST). Many studies using the WCST have shown differences in behavioral patterns between individuals with ASD and their controls (Hill, 2004a,b; Geurts et al., 2009).

Wisconsin card sorting test is a neuropsychological test, in which different cards need to be sorted on one of three possible dimensions (color, number, or shape). The currently correct dimension is not explicitly announced and changes according to a fixed number of trials. The participant receives feedback when the card is placed incorrectly. Based on this feedback, the participant needs to make a decision whether to continue or to change their sorting rule. The performance on WCST is measured in terms of errors, and individuals with ASD tend to make more perseverative errors compared to typically developing individuals (Goldstein et al., 2001; Geurts et al., 2004; Lopez et al., 2005; Tsuchiya et al., 2005; Verté et al., 2005, 2006). These errors are seen as a failure to shift to the new sorting rule and, therefore, as an index of cognitive inflexibility. Accordingly, the major conclusion drawn from these studies is that the tendency for highly perseverative responses to the WCST observed in individuals with ASD reflects their problems with changing the focus, switching the tasks, and hence difficulties in cognitive flexibility.

Intriguingly, however, when tested in a more controlled experimental settings, this idea of deficits in cognitive flexibility as measured by deviant task switching performance is hardly supported by any empirical evidence (Stahl and Pry, 2002; Schmitz et al., 2006; Whitehouse et al., 2006; Shafritz et al., 2008; Dichter et al., 2010; Poljac et al., 2010; Hayward et al., 2012). For instance, Hayward and colleagues used stimuli that included global shapes (diamonds, squares, circles) that were made up of smaller elements of the same shapes. This combination of global and local elements allowed the authors to test performance flexibility in a group of high-functioning young adults with ASD and in their controls. Flexibility was measured as a difference in performance (speed and accuracy) between two conditions: the condition in which participants responded either to the global or to the local level of the stimulus element with the condition that included switching between the two levels. The authors observed similar patterns of behavior regarding flexibility between the two groups and hence no empirical evidence for deviant flexibility in individuals with ASD. A comparable finding was observed in children and adolescents with ASD in experimental conditions that required them to switch between tasks that were explicitly specified (Dichter et al., 2010; Poljac et al., 2010). These observations of no deviant flexibility in ASD reported in studies using more experimentally controlled tasks are in sharp contrast to clinical studies and reports on daily lives of individuals with ASD. As already pointed out by Geurts et al. (2009), this discrepancy between the observed behavioral rigidity and the lack of empirical support for cognitive inflexibility raises the question of how to resolve this paradox. The authors propose four different points to take into account when trying to specify the cognitive mechanisms behind the behavioral inflexibility in ASD. First, to help us address the comorbidity often present in ASD and specify the differences and similarities between ASD and other neurodevelopmental disorders, the studies would need to be designed such that the behavior of the individuals with ASD is compared not only to a typically developing comparison group but also to comparison groups involving other neurodevelopmental disorders. Second, studies with larger sample size would also be desirable to validate the non-significant group differences reported in studies with small sample size. Third, the authors suggest the use of mechanistic and detailed measures that are theory based – to help us specify the cognitive deficits in ASD – as well as the use of ecologically valid measures – to help us link the actual behavior to task performance. Finally, the authors suggest that other top-down (social-motivational) and bottom-up (arousal and stress) factors than discussed so far also need to be considered when trying to explain inflexibility in ASD. Other studies on cognitive control – usually involving typically developing individuals – have already shown that both top-down and bottom-up factors strongly determine our goal-directed behavior (Kiesel et al., 2010; Vandierendonck et al., 2010).

### Goal-directed behavior and cognitive control in ASD

Goal-directed behavior has been investigated within different research fields, such as those investigating task switching and action control. These studies typically reflect scientific attempts to understand neurocognitive control mechanism behind goal-directed behavior, both in terms of the formation of higher-order task intentions as well as in terms of their implementation and specific translation to thought and action. Intentions can be defined as tendencies that guide our interaction with the environment and can be specified at different levels (Ondobaka et al., 2012). Although the categorization of the levels might differ between research fields, we could for instance define intentions at higher-order goal level (e.g., quench the thirst) as well as at the action (e.g., drink some water) and the motor level (e.g., grab a glass of water; for an interesting discussion on this topic, see Uithol et al., 2012).

As we know from different lesion studies, this ability to exert intentional control is not self evident (Aron et al., 2004). Also some empirical evidence for difficulties in intentional control have been reported in clinical populations, like for instance delayed experience of intention in GTS (Moretto et al., 2011) or the imbalanced relation between voluntary and automated modes of control in PD (Torres et al., 2011). Furthermore, deficits in intentional resistance to interference have been reported in individuals with schizophrenia (Paulik et al., 2009). The processes involved in intentional resistance to interference that is introduced in experimental settings by presenting task irrelevant information *simultaneously* with the task relevant information relates to the ability of the cognitive system to protect the current task execution. Intentional resistance to interference differs from proactive type of interference that arises from *previously* relevant task information – the process that is not directly related to intentional control – but resembles strongly the processes of intentional inhibition that has been reported to be impaired in individuals with ADHD (Roberts et al., 2011) and also in children with ASD (Christ et al., 2011). In individuals with ASD, just a few studies have been conducted so far that investigated their intentional control, for which task switching procedures were applied (Schmitz et al., 2006; Whitehouse et al., 2006, Experiment 3; Poljac et al., 2010). The rationale behind studying intentions with rapid task switching procedures is the assumption that our ability to perform instructed tasks is a clear expression of intentional control. These studies have not provided any indication of a deviant task switching behavior in ASD or problems with intentional control. From the studies on action control, however, some empirical evidence has been provided for problems at the level of translation of intentions into actions (Cattaneo et al., 2007; Boria et al., 2009). Cattaneo and colleagues have for instance shown that children with ASD show a deficit in predicting the final goal of an action sequence based on the initial or antecedent motor act. Despite the fact that instructed task switching studies so far indicate no clear deficits in intentional control, and a few studies on action control indicate the problems at the level of translation of intentions into actions, we will try and elaborate more on the possibility of deficits in intentional control in ASD. This idea is especially interesting to consider given the usually reported inability of individuals with ASD to generate novel ideas and behaviors spontaneously (Boucher, 1988; Craig and Baron-Cohen, 1990; Turner, 1999; Wong et al., 2003) and difficulties with shared intentionality (cf. Tomasello et al., 2005).

## Intentional and Motor Control in ASD

### Instructed and voluntary task switching studies

Being able to direct our thoughts and actions allows us to adaptively respond to changes in internal and external conditions. Clearly, intentional control provides the basis for cognitive flexibility. Interestingly, however, recent studies on task switching have reported empirical evidence for a small but consistent preference for repetitive voluntary behavior in typically developing individuals. Specifically, when given an option to choose between two tasks and deliberately decide which task to perform in each trial, typically developing individuals show a tendency to repeat tasks more often than to switch between them (Arrington and Logan, 2004; Mayr and Bell, 2006). This repetition bias occurs even though people are explicitly instructed to choose tasks at random and equally often.

Voluntary task switching procedures are developed to address the issue of task intentionality in procedures in which the tasks are cued – externally defined – in each trial (Arrington and Logan, 2004, 2005). More precisely, the question has been raised as to how much of intentional control one needs to exert if the tasks are (pre)specified. Certainly, these instructed procedures offer much more controlled way of testing cognitive control than complex neurocognitive tests, such as the WCST. However, we also know from different studies that task switching behavior is strongly affected by task history. This observation is usually explained in terms of bottom-up effects. Specifically, a carry-over of activation from previous tasks is suggested to modulate the execution of the current task (Allport et al., 1994; Sohn and Carlson, 2000; Wylie and Allport, 2000; Ruthruff et al., 2001; Monsell, 2003). It is therefore possible that when task execution is based on explicitly given task instructions, the processes involved reflect some other control mechanisms than those involved in intentional control. For instance, behavior measured in these conditions might reflect processes needed to resolve proactive interference from previous tasks rather than reflecting any kind of intentionality. This possibility is in particular interesting for understanding cognitive control mechanisms in ASD, since Christ et al. (2011) have recently shown that children with ASD demonstrated no difficulties with resolving proactive interference.

In voluntary task switching procedures, the intentional component is separated from the subsequent action: Whereas the choice of tasks is assumed to reflect intentional control, the subsequent task execution would reflect the translation of intentions into the corresponding actions. Separating task choice from task execution allows us to disentangle the participants’ global task intentions from their specific actions. Different studies have provided evidence that these two reflect related yet dissociable processes (Mayr and Bell, 2006; Lien and Ruthruff, 2008; Arrington and Yates, 2009; Yeung, 2010; Butler et al., 2011; Orr and Weissman, 2011; Poljac et al., 2012). Specifically, while we know that task choice and task execution are sensitive to similar influences (Lien and Ruthruff, 2008; Yeung, 2010; Orr and Weissman, 2011), we also know that the expression of the behavioral costs related to task choice and those related to task execution differ. This difference becomes perhaps most clearly evident when using tasks that differ in their relative strength: While it takes longer to make a choice to switch to the more difficult task, performance costs are larger when switching to the easier task (Millington et al., in revision). Another interesting finding related to differences in relative task difficulty is that it modulates people’s tendency to repeat tasks such that people tend to exhibit a stronger repetition bias for the relatively harder task.

Voluntary task switching has recently been investigated in typically developing participants with either a high or a low level of autistic traits (Poljac et al., 2012). Autistic traits were assessed by a self-report questionnaire that quantifies the extent of autistic traits in healthy population – the Autism spectrum Quotient (AQ). The AQ has been used extensively to investigate the BAP with converging evidence that autism is not just a spectrum within the clinical population, but that autistic traits are continuously distributed through the general population (Baron-Cohen et al., 2001; Hoekstra et al., 2007). As often in studies using voluntary procedures, Poljac and colleagues required the participants to switch voluntarily between two tasks, while encouraging them to choose the tasks at random and equally often. In each trial, participants first indicated to have made a choice between a relatively easy location and a relatively hard shape classification task by pressing a space bar. After having made a task choice, they then responded to the subsequently presented stimulus. Consistent with previous studies with instructed task procedures in ASD (Schmitz et al., 2006; Whitehouse et al., 2006; Poljac et al., 2010), no difference was observed between the high and the low AQ participants in the behavioral patterns of *task execution*. The authors reported that both groups replicated in a similar way earlier findings regarding task execution reported in studies using voluntary task procedures by exhibiting reliable switch costs (Arrington and Logan, 2004) and reliable asymmetry in switch costs (Yeung, 2010). Importantly, however, significant differences in *task choice* were observed between the two groups: The asymmetry in repetition bias – typically observed in healthy individuals – was pronounced more strongly in individuals with high AQ scores, as they showed a higher tendency to repeat the harder task more often than the individuals with low AQ scores. These findings of similarities in task execution but differences in task choice between the two groups seem to suggest that repetitive behavior in ASD is possibly associated with processes involved in the formation of general task intentions rather than with processes involved in implementation of these intentions and their translation to the corresponding actions. While the study clearly provides first indication of deviant intentional control but intact control of task execution in ASD, this idea needs to be tested further in the clinical population. This might help us specifying the mechanism behind the observed repetitive behaviors and possibly also behind the cognitive inflexibility in ASD.

### Action control and anticipating intentions of others

Investigating how people control their actions while interacting with the world in a goal-directed manner has provided empirical evidence that people strongly rely on sensory information for making predictions of events in the environment. In fact, it has been suggested that learning to make new movements goes together with building of associations in our brain between actions and sensory feedback. This allows us to predict the sensory consequences of self-generated action and to maximize performance in a given environment (Izawa et al., 2008). This close link between action and perception is for instance supported by findings that people tend to move their eyes toward the object that they want to act upon in advance of their actual movement (Ballard et al., 1995; Epelboim et al., 1995; Land et al., 1999; Johansson et al., 2001; Sailer et al., 2005). It seems that these anticipatory eye movements aid the control of goal-directed behavior when visual input is available. Interestingly, observing others performing actions elicits similar eye movement patterns to those elicited when people execute the actions themselves both in adults (Flanagan and Johansson, 2003; Rotman et al., 2006) and in infants (van Elk et al., 2008; Rosander and von Hofsten, 2011). Action execution and action observation seem also to elicit activation of neurons in specific and to some extent overlapping brain areas (di Pellegrino et al., 1992; Gallese et al., 1996; Rizzolatti et al., 1996). These mirror neurons were originally discovered in the ventral premotor cortex of the macaque monkey (area F5). In humans, a similar circuit has also been discovered, involving parieto-frontal network (Rizzolatti and Craighero, 2004). It has been suggested that the functional role of the mirror neurons is to encode motor acts and movements, subserving the understanding of the motor intention underlying the actions of others, without providing information about the reasons why a certain action takes place (Rizzolatti and Sinigaglia, 2010; for a critical note, see Hickok, 2009).

Investigation of motor learning and action control in ASD has recently provided evidence that children with ASD rely stronger than normal on proprioceptive feedback when learning new movements (Haswell et al., 2009; Izawa et al., 2012). This observation is important as it suggests abnormalities in the processes underlying the formation of perception-action associations in ASD. The ability to predict the sensory consequences of self-generated action is crucial for optimizing our goal-directed behavior. According to Friston (2010), for instance, people optimize their goal-directed behavior using sensory inputs such that they maximize the expected rewards and minimize the expected costs. Deviations in this predictive mechanism would be reflected in performance on tasks requiring goal-directed actions. Consistent with this idea, empirical evidence has been provided for deficits in predicting the final goal of a complex action in children and adults with ASD (Cattaneo et al., 2007; Zalla et al., 2010a). For instance, when required to reach and grasp a piece of food and bring it to the mouth, children with autism showed different pattern of food anticipation as measured by the activation of the mylohyoideus muscle (MH). Specifically, Cattaneo and colleagues showed that while the MH activation increased in typically developing children much before their hand grasped the food, the MH activation in children with ASD did not increase before the last step of the complex action, that is, not before they started moving the hand holding the piece of food toward the mouth. Boria et al. (2009) further specified this deficit in action prediction by providing evidence that rather than having problems with predicting the action itself (what is done), children with ASD found it difficult to predict the intention behind the action (why is it done). Furthermore, it seems that children with autism have a deficit in integrating motor acts into a global action (Fabbri-Destro et al., 2009). Related to these observations of deficits at the level of motor learning and action control (for structural brain deviations, see Mostofsky et al., 2007), different studies have proposed that individuals with ASD experience problems with understanding the action intentions of others due to mirror neurons (Cossu et al., 2012; for a review see Iacoboni and Dapretto, 2006). These neurological deviations in the parieto-frontal mirror neuron circuit are considered to be at the basis of their problems in social interactions (Ramachandran and Oberman, 2006; Oberman and Ramachandran, 2007), although some scientists challenge this view (Southgate and Hamilton, 2008; Hamilton, 2009).

Another interesting point to address here is that most of the studies testing action control and intentions in ASD so far, have applied repeated presentation of the same stimulus material over short period of time, often with meaningless and simple actions. Using meaningful multistep actions and dynamical stimulus material instead (auditory stream and video material; cf. Carmi and Itti, 2006) would make it possible to measure anticipatory eye movements in an experimental paradigm that better represents how people usually interact with the real world. Such a paradigm would allow for differentiating between single action steps and the final step (goal) of an action sequence. Recently empirical evidence has been provided that anticipatory eye movements in typically developing individuals are elevated for the last and the final step in action sequences consisting of three action steps (Poljac et al., in revision). The action sequences were applied in two different but highly comparable paradigms – one relying more on action control and the other on language comprehension. Interestingly, this pattern of increased amount of predictive looks for the final action step was similar for both the action and the language paradigms. These observations imply that anticipatory eye movements reflect the working of a shared predictive mechanism that accumulates semantic information relevant for our final (motor or linguistic) behavioral goal in complex action sequences.

Interactions with the world, and in particular those involving other people, would clearly lack fluency without such a predictive mechanism. It facilitates our understanding of a given situation, allowing for efficient and adaptive interaction with the environment. Even social interactions that at surface look easy – walking next to each other or having a conversation – would be quite difficult to perform fluently and timely if our brain would exclusively rely on slow feedback information processing. Obviously, for a joint (motor or linguistic) activity to be successful, interpreting each other’s actions or words after they occur would not be sufficient. Considering the fact that one of the core symptoms of ASD is their problems in social interactions and communication, it would be quite interesting to test if individuals with ASD would demonstrate the accumulation of the predictive looks in the final step of complex action sequences, both in the action and in the language paradigm. If the predictive mechanism is generally impaired in ASD, then the level of predictive looks would be expected to be overall lower in individuals with ASD than in typically developing individuals. If, however, the predictive mechanism is impaired due to deficits related to intentional control, then one would expect to observe around an equal amount of predictive looks across the whole action sequence without an increase in anticipatory eye movements in the final step.

Interestingly, also in the domain of action control and intentions, the observed difficulty with predicting – in this case the anticipatory postural adjustments (Schmitz et al., 2003; Vernazza-Martin et al., 2005) – seems to be predominantly present for actions with voluntary movement onsets, rather than in conditions when the onset was imposed (Martineau et al., 2004). This finding is remarkable as it is in line with the suggestion from the task switching studies that not the instructed (Schmitz et al., 2006; Whitehouse et al., 2006; Geurts et al., 2009; Poljac et al., 2010) but the voluntary type of task switching (Poljac et al., 2012) seems to be difficult for individuals with ASD. We will further discuss this observation in the context of a recently proposed idea of open and closed systems (Lawson, 2003; Lawson et al., 2004) in the following section.

## Cognitive Control in Open and Closed Tasks

Investigation of the intentional control mechanisms in ASD within both cognitive flexibility (task switching studies) and action control (perception-action studies) have provided empirical indications that problems predominantly arise in tasks of an open character rather than in tasks that are more strictly defined. Individuals with ASD seem to be able to follow the instructions and flexibly switch between tasks when tasks are clearly specified but not when the tasks leave space for interpretation (cf. Klin et al., 2003; Geurts et al., 2009). Indication for particular problems with voluntary task choices (Poljac et al., 2012) supports this notion and suggests problems at the intentional level rather than at the level of the implementation of intentions. In a similar fashion, evidence coming from action control suggests deficits at the intentional level (Boria et al., 2009) and in particular for those situations that involve voluntary action control (Martineau et al., 2004).

Interestingly, also in language and memory, empirical evidence has been provided for this distinction between mostly problematic voluntary (user driven) control and usually unimpaired instructed (stimulus driven) control. For instance, children with ASD experience difficulties in mapping novel words onto unnamed entities if they need to rely on speakers’ referential intentions (Baron-Cohen et al., 1997), but not if the amount of objects without a name is restricted (Preissler and Carey, 2005). This restriction of objects when mapping new words to novel objects in the world helps young children with ASD to learn meanings of new words equally successfully as healthy controls. Also, studies examining memory functioning in ASD (see Goh and Peterson, 2012 for a recent review on neuroimaging findings on learning an memory deficits in ASD) showed that, while paradigms testing cued-recall of words tend to demonstrate no deviant performance (Bowler et al., 1997), free recall paradigms generally lead to diminished performance in this population (Smith et al., 2007; Gaigg et al., 2008). Also when tested for free recall of actions, individuals with ASD differ from typically developing individuals as they seem not to benefit from the enactment effect, that is, the memory enhancement for enacted compared to observed actions (Zalla et al., 2010b). Furthermore, children with ASD find it difficult to memorize task rules based on arbitrary (learned in the lab) stimulus-response (S-R) associations but not memorizing task rules based on straightforward (learned outside the lab) S-R associations (Stoet and López, 2001). Accordingly, adults with ASD experience difficulties with novelty processing of stimulus material (Maes et al., 2011). All these observations are in line with the challenges that individuals with ASD experience when generating novel ideas and behaviors spontaneously (Boucher, 1988; Craig and Baron-Cohen, 1990; Turner, 1999; Wong et al., 2003). An interesting idea has been proposed to explain the difference in task success between tasks that are loosely defined and those that are strictly defined in their demands – individuals with ASD would- according to this view have difficulties with “open” type of systems (Klin et al., 2003; Lawson, 2003; Lawson et al., 2004; White et al., 2009).

### Open and closed systems

Real-life situations typically involve huge variety in options for our interactions with the environment and with other people. Handling these situations optimally often requires highly adaptive human behavior. One could say that our everyday interactions with the world with many (if not endless) degrees of freedom involve a system that is open in how a specific situation will eventually be solved by an individual. An *open* system is the one that cannot be reduced to one predefined solution of a given situation. *Closed* systems, on the contrary, are those for which we know the exact output given a certain input (Bhaskar, 1978; Winograd and Flores, 1986; Klin et al., 2003; Lawson, 2003; Lawson et al., 2004).

### Open and closed tasks in ASD

Typically developing individuals experience the world as an open system with unpredictable relations between the events, reflecting a fairly accurate view of the actual world. Individuals with ASD, on the contrary, experience their environment with all its elements as a closed system. Such an attitude toward world consists of definite and predictable event regularities. Accordingly, this mode of thinking disregards irregularities, the complexity and unpredictability of situations. Such a conception would generate difficulties in coping with dynamics of daily life possibly reflected in behavior as repetitive and rigid responding to the environment. Similarly, this cognitive style would generate difficulties in coping with experimental tasks that are more ecologically valid, that is, those tasks that better reflect real-life situations (Klin et al., 2003; Kenworthy et al., 2008). Therefore, neuropsychological tests and experimental tasks that are more complex, such as WCST and voluntary task switching procedures, would be more sensitive to detect these differences in cognitive styles (White et al., 2009).

This rather descriptive explanation for the observed discrepancies between the behavior inside and outside the lab is valuable as it provides a framework for further investigation of different inconsistencies (Geurts et al., 2009; Teunisse et al., 2012) in findings on cognitive control in ASD within different research areas. It would be interesting to see if we can specify the neurocognitive mechanism behind the differences in behavioral patterns observed between open and closed tasks. More precisely, it would be informative to test if the processes that generate these differences could be specified by leveraging our knowledge on intentional control difficulties in ASD (Baron-Cohen et al., 1997; Martineau et al., 2004; Boria et al., 2009; Poljac et al., 2012). For instance, one could directly test whether these differences in cognitive styles as described in terms of predictability of event regularities (open vs. closed systems) are predictive of someone’s ability in intentional control. An interesting note to this part is that Klin et al. (2003) have for instance already used this intriguing observation of discrepancies in behavior in clearly instructed and more natural situations as a starting point to explain difficulties that individuals with ASD experience in social interactions. The authors put forward an idea that human cognition is embedded in experiences resulting from a body’s actions upon salient aspects of its surrounding environment. The way this occurs in ASD differs from early on in the development, starting with differences in predisposition for stimulus saliency. Because of this, babies with autism develop a different way of processing sensory information, which in turn defines the way that they interact with the environment and hence determines what it is that they build their experiences on. This leads to the possibility that social world as enacted by individuals with ASD is most probably quite different from that enacted by typically developing individuals. This idea emphasizes the importance of the development of cognition. The developmental approach to understanding cognitive control in ASD predominantly relies on studies involving children and some studies involving adults, while studies focusing on elderly with ASD are rare (Geurts and Vissers, 2012; Happé and Charlton, 2012). As different studies have shown, capacities in cognitive control vary across age in typically developing individuals (Cepeda et al., 2001; Kray et al., 2004; Crone et al., 2006; Davidson et al., 2006). In the following section, we will therefore address the possible connections between the development of cognitive control mechanisms across age in typically developing and in individuals with ASD.

## Cognitive Control Across Age

### Typically developing individuals

Various studies have examined how the ability to exert control over our thoughts and actions differs for different age groups in typically developing individuals (Zelazo et al., 2004; Crone et al., 2006; Meyer et al., 2010; Washylyshyn et al., 2011; Yeniceri and Altan-Atalay, 2011) with just a few longitudinal studies so far, a genetic study (Erickson et al., 2008) and two neuroimaging studies (Finn et al., 2010; Koolschijn et al., 2011). The idea behind this developmental approach is based on the observations that different cognitive control abilities change throughout the life. The change is usually such that the abilities first improve between infancy and late adolescence (Luciana and Nelson, 1998; Diamond, 2002; Zelazo et al., 2004) followed by their deterioration later on in life (Kray and Lindenberger, 2000; Cepeda et al., 2001; Reimers and Maylor, 2005).

In a similar fashion, studies on cognitive flexibility as measured by the WCST have indicated changes in perseverative behavior across age (for developmental perspective on executive functions in ASD, see Russo et al., 2007). Despite the consensus on the finding that perseveration decreases with age, the exact age at which perseveration on the WCST attains adult levels is still under debate: while some studies have indicated that children exhibit patterns of behavior on perseverative errors similar to adults by age 10 (Chelune and Baer, 1986; Welsh et al., 1991), other indicate age 14–15 (Crone et al., 2004) or that the perseverative performance even continues developing into young adulthood (age 21, Huizinga et al., 2006). The perseverative behavior increases again later in life (Rhodes, 2004). Older adults usually commit more perseverative errors than younger adults (Axelrod and Henry, 1992; Fristoe et al., 1997; Ridderinkhof et al., 2002; but, see Haaland et al., 1987). From all these studies, it seems that cognitive flexibility as assessed by the WCST demonstrates an inverted U curve, but that the exact development across age is hard to specify as it strongly varies across different studies.

Task switching literature has also provided rather complex and often inconsistent age related differences in cognitive control capacities. In these studies, a distinction is usually made between switch costs and mixing costs. Switch costs, as described earlier, are calculated as a difference in behavior between switch and repeat trials. Mixing costs, on the other hand, are usually calculated for task repetitions only, reported as slower responses on repetition trials under mixed-tasks conditions than under single-task conditions (Los, 1996; Koch et al., 2005; Rubin and Meiran, 2005; Steinhauser and Hubner, 2005; Poljac et al., 2009). In developmental studies, however, mixing costs are often referred to as global costs and are calculated as differences in behavior between repetitions in single-task conditions and the average of repetition and switch trials in mixed-tasks conditions. Possibly due to these differences in definition, varying results have been reported for mixing costs and their relation to age. When calculated as repetitions only, some studies have reported no differences across age (Span et al., 2004; Crone et al., 2006). Crone et al. have, for instance, observed a stable pattern for mixing costs across their three age groups: 7–8; 10–12; and 20–25 years old. On the contrary, when calculating mixing costs including switch trials, larger mixing costs are reported in children than in adults (Cepeda et al., 2001; Kray et al., 2004; Reimers and Maylor, 2005). Kray et al. have also reported an inverted U curve for mixing costs, with both children and elderly exhibiting larger mixing costs than young adults.

Switch costs usually show a stable pattern across age when general age related slowing is taken into account (Brinley, 1965; Salthouse et al., 1998; Kray and Lindenberger, 2000; Mayr and Kliegl, 2000; Mayr, 2001; Reimers and Maylor, 2005), however, not always (Van Asselen and Ridderinkhof, 2000; Kray et al., 2002). For instance, Van Asselen and Ridderinkhof reported that older adults demonstrated significantly greater switch cost than young adults when task switches were unpredictable but not for the predictable ones. This suggests that older adults were also able to exhibit similar cognitive flexibility if they were given an opportunity to prepare for a task switch (cf. Kramer et al., 1999; Kray and Lindenberger, 2000).

Although the exact turning points into maturation and the later deterioration of the cognitive control mechanisms involved in flexible cognition are difficult to specify from the literature so far, the age related dependences of both perseverative as well as on switching behavior are evident. Surely, differences in definitions make it harder to compare different studies. In addition, because children are often incapable of managing with adult tasks, different tasks are used for different ages, making the comparison across age quite difficult (see Zelazo and Mueller, 2002 for a list of different tasks used to test cognitive control in children). Even more importantly, however, a question that remains- when it comes to this part of the literature examining cognitive control in healthy population- is what exactly is causing the detected age-related differences. Crone et al. (2006) have suggested that differences in task switching behavior across age might arise due to differences in carry-over effects of S-R associations between trials. According to Crone and colleagues, younger children might build stronger S-R associations that lead to a strong bias toward the same response if the stimulus repeats across trials. In a similar way, Mayr (2001) suggested that his finding of less flexibility in older adults might also be due to stronger carry-over effects from previously formed S-R associations. Interestingly, influences from traces of past tasks are also shown to influence task performance in individuals with ASD (Zmigrod et al., 2012). In what follows, we will discuss the few studies conducted so far on cognitive control development in ASD.

### Individuals with ASD

Autism spectrum disorders is generally understood as a pervasive, lifelong condition (American Psychiatric Association, 1994). It is quite surprising then that there have been just a few studies tracking the development of cognitive (dis)abilities in ASD and that, despite the general wealth of research in ASD. One would expect that any putative explanation of the observed behavioral patterns specific to ASD, such as difficulties in intentional control or need for sameness, should be able to account for the (dis)continuities and that take place across the life span.

The few longitudinal studies on cognitive control in ASD conducted so far have provided empirical evidence for a stable deficit in flexibility. For instance, Ozonoff and McEvoy (1994, Experiment 1) have reported in their 3-year follow-up study that while the control group of adolescents with learning disability showed a reduction in perseverative behavior on the WCST, concomitant improvements were not evident in adolescents with ASD. Using spatial reversal task that also measures perseverative behavior, Griffith et al. (1999) reported similar stable perseverative behavior in preschoolers with ASD within the space of 1 year. Also a more recent longitudinal study conducted by Pellicano (2010) confirmed that children with ASD were impaired in their cognitive flexibility relative to their matched controls at both time points that the testing occurred. These studies demonstrated a rather stable perseverative behavior, implying that there might be a ceiling on the extent to which such cognitive abilities can develop in individuals with ASD. Pellicano has, however, provided evidence that higher-order planning – measured by the ToL task – improved significantly more in children with ASD than in the typically developing controls. This finding implies that although deficits in cognitive flexibility seem to show a stable pattern over the development in individuals with ASD, some other cognitive functions that are also important for behavioral control might change with age.

More developmental studies are evidently needed to be able to specify how this developmental and lifelong disorder manifests in behavior across the life span. For instance, more studies involving elderly with ASD would generally be desirable (cf. Geurts and Vissers, 2012; Happé and Charlton, 2012). More specifically, studies are needed that would help us determine the age at which we can start differentiating between cognitive control capacities in typically developing and individuals with ASD (for early brain development in ASD, see Courchesne et al., 2007). Also studies specifying further development of the detected cognitive control deviations would be valuable for our better understanding of autism. It would for instance be interesting to see how both cognitive flexibility and higher-order planning are reflected in behavior of elderly with ASD.

An interesting parallel to consider would be to investigate how action control and planning are affected by age in ASD. Some recent developmental studies have indicated a substantial improvement in interactive action control in typically developing children between the age of 2.5 and 3 (Meyer et al., 2010). Specifically, while 3-years old children were able to demonstrate a pattern similar to adults for both individual and joint actions, children of 2.5 demonstrated less accurate performance when acting jointly with an adult partner. Considering the fact that individuals with ASD show problems in social interactions – for instance, understanding emotions of others (Dapretto et al., 2006; Ebisch et al., 2010) and collaborative engagement seem to be impaired in children with ASD (Carpenter et al., 2005; Tomasello et al., 2005) – it would be interesting to test if we can specify the age at which these problems are reflected in behavior and how these further develop throughout their life.

Finally, if future studies empirically confirm deficits at the level of intention establishment in ASD, it would be interesting to see if any developmental component could be related to impulsive, compulsive, and apathetic behavior often reported in individuals with ASD. It is plausible to expect that a cognitive system lacking intentional control would be a system, in which most of the (external and internal) stimulation is being processed. In other words, it would be a system that impulsively responds to any incoming stimulus. As we know that our brain is highly plastic (for a recent review, see Zimmerman and Lahav, 2012), we would expect that, during the development, the brain would compensate for this overload on information. A possible way to do so is by focusing on a single, highly preferred intention, a strategy that might result in behavioral compulsivity. The brain might perhaps even overcompensate, resulting in a system that is predominantly shut to the most of the stimulation, such is the case in apathy. We would expect that impulsivity, compulsivity, and apathy are interrelated and follow each other across the development of the brain of individuals with ASD as adaptive compensations to the differently wired brain from the start on (cf. Klin et al., 2003).

## Conclusion

### What have we learned so far?

In this review, we summarize and critically reflect on behavioral findings and their theoretical explanations regarding cognitive control processing in autism, also from a developmental perspective. The specific focus of this review is the recent evidence of deficits in intentional control – a specific subset of cognitive control processes that biases the choice of our behavioral goals – coming from different research fields. We relate this evidence to the cognitive rigidity observed in ASD and argue that individuals with ASD experience problems at intentional level rather than at the level of the implementation of intentions. Both these processes are related to cognitive control mechanisms but in different ways. Finally, we discuss new directions in studying cognitive control in ASD and how these relate to adaptive cognition.

This review focuses on cognitive control impairments in ASD. We conclude from the literature we have summarized and discussed that although the deficits in cognitive control in individuals with ASD have received a considerable amount of empirical support at the behavioral level, the specific neurocognitive mechanisms behind the observed behavioral deviations are still heavily debated. One of the challenges we need to address is the observation that the behavior of individuals with ASD in the lab often differs from that observed in real-life or in clinical settings. This is for instance the case with cognitive flexibility in ASD – while behavioral rigidity is evident in their daily lives, testing the assumed difficulties in cognitive flexibility in ASD by means of task switching paradigms provides hardly any empirical evidence (Geurts et al., 2009). First steps in addressing this challenge are already set by providing a more detailed description: while complex neuropsychological tests are predominantly ambiguous in their output demands and are hence open to various interpretations of the required behavior, the more controlled experimental settings are quite precise in their behavioral demands (Lawson, 2003). The reported difficulties with neuropsychological tests would according to this view reflect the tendency of individuals with ASD to approach the world in a more strictly defined relation between internal and external input and their responding to the same (cf. Klin et al., 2003). Finally, we elaborate on this observation and highlight the possibility that individuals with ASD predominantly exhibit problems with tasks of an open character rather than in tasks that are clearly defined due to their deficits in intentional control. Specifically, the idea we put forward in this review is that cognitive control deficits in ASD are generated at the level of intentional rather than executional processing. Deficits in intentional control would go together with difficulties in adaptive behavior, which is typically observed in individuals with ASD.

### Is cognitive control approach a promising way to follow

Clinical and behavioral studies have predominated the research on ASD for quite some time (cf. Hill and Frith, 2003; Frith and Happé, 2005), possibly encouraged by the fact that its diagnostic criteria as described in DSM IV are based on behavioral expression of this neurodevelopmental disorder (see e.g., Robbins et al., 2012 for a critical view on DSM IV). The recent increase in neuroimaging (for review, see Belger et al., 2011; Philip et al., 2012) and genetic (for review, see Eigsti and Shapiro, 2003; Geschwind, 2011) studies has certainly significantly contributed to our knowledge of ASD – we know now that the mechanisms behind the observed deviant behavioral patterns are not related to straightforward gene deviations or a simple brain deficit.

The complete understanding of ASD will obviously require a better understanding of how genetic risk variants lead to changes in neural circuitry and function and their expression in covert and overt behavior. From the cognitive approach, the challenge is to relate specific deficits at the brain level, via more general descriptions at a cognitive level (i.e., cognitive control impairments) to the reported behavioral differences (Frith, 1998, 2012). Specifically, cognitive control deficits as expressed in behavior of individuals with ASD need further specification at the level of neurocognitive mechanisms. This review concludes that within the proposed theoretical approach of executive dysfunctions, a more precisely defied approach needs to be offered if we are to take the challenge of specifying the individuals within the ASD population at the genotype and phenotype level in more detail.

We propose here that a promising way to follow would be to systematically explore the recently reported indication of deficits in cognitive control in ASD at the level of higher-order intentions, coming both from research field on cognitive flexibility (task switching studies) and on action (perception-action studies). Finally, studies that would systematically compare intentional control deficits in ASD with intentional deficits reported in other neuropsychological disorders – such as schizophrenia and GTS – are desirable and interesting as they might reveal if these disorders may share some underlying cognitive mechanisms with ASD.

## Conflict of Interest Statement

The authors declare that the research was conducted in the absence of any commercial or financial relationships that could be construed as a potential conflict of interest.
